# Primary Thromboprophylaxis in Ambulatory Cancer Patients: Where Do We Stand?

**DOI:** 10.3390/cancers12020367

**Published:** 2020-02-05

**Authors:** Frits I. Mulder, Floris T. M. Bosch, Nick van Es

**Affiliations:** 1 Department of Vascular Medicine, Amsterdam Cardiovascular Science, Amsterdam UMC, University of Amsterdam, 1105 AZ Amsterdam, The Netherlands; f.t.bosch@amsterdamumc.nl (F.T.M.B.); n.vanes@amsterdamumc.nl (N.v.E.); 2 Department of Internal Medicine, Tergooi Hospitals, 1213 XZ Hilversum, The Netherlands

**Keywords:** venous thromboembolism, cancer-associated venous thromboembolism, thrombosis, pulmonary embolism, neoplasms, anticoagulants, direct oral anticoagulants, coumarins, low molecular weight heparins

## Abstract

Venous thromboembolism (VTE), comprising deep-vein thrombosis and pulmonary embolism, is a frequent complication in ambulatory cancer patients. Despite the high risk, routine thromboprophylaxis is not recommended because of the high number needed to treat and the risk of bleeding. Two recent trials demonstrated that the number needed to treat can be reduced by selecting cancer patients at high risk for VTE with prediction scores, leading the latest guidelines to suggest such an approach in clinical practice. Yet, the interpretation of these trial results and the translation of the guideline recommendations to clinical practice may be less straightforward. In this clinically-oriented review, some of the controversies are addressed by focusing on the burden of VTE in cancer patients, discussing the performance of available risk assessment scores, and summarizing the findings of recent trials. This overview can help oncologists, hematologists, and vascular medicine specialists decide about thromboprophylaxis in ambulatory cancer patients.

## 1. Background and Aim

Venous thromboembolism (VTE), comprising deep-vein thrombosis and pulmonary embolism, is a frequent complication in cancer patients. Overall, approximately 8% of patients develop VTE in the first year after their cancer diagnosis [1]. However, the risk of cancer-associated VTE heavily depends on tumor type, ranging from 1% in patients with low-risk tumors, such as breast or prostate cancer, to up to 20% in those with high-risk tumors, such as pancreatic cancer [2]. Other important risk factors include tumor stage and chemotherapy [3,4]. 

Despite the high risk, routine thromboprophylaxis for all ambulatory cancer patients is not recommended [5,6]. However, recent studies have brought new insights leading to changes in guidelines on primary VTE prevention in cancer patients [7,8]. Yet, the interpretation of these trial results and the translation of the guideline recommendations to clinical practice may be less straightforward. In this clinically-oriented review, we will address some of the controversies to help oncologists, hematologists, and vascular medicine specialists in making decisions about thromboprophylaxis in ambulatory cancer patients. We will focus on the burden of VTE in cancer patients, discuss several of the available risk scores, summarize the findings of recent trials, and provide potential directions for future research.

## 2. The Burden of Venous Thromboembolism in Cancer Patients

Cancer patients often experience complications during the course of their disease, including infections, side effects of chemotherapy, and symptoms directly related to the tumor. VTE is yet another frequently occurring complication. However, the decision to provide thromboprophylaxis to cancer patients has to depend on a careful assessment of the benefits (i.e., reduction in VTE and possibly arterial thromboembolism) and harms (i.e., bleeding). To be able to weigh the risks and benefits, clinicians need to be familiar with the burden of VTE in cancer patients. We will now summarize the available data on the short- and long-term consequences of VTE, which are listed in Table 1.

### 2.1. Mortality

The primary goal of physicians treating cancer patients is to prevent death. It has been well recognized that VTE is strongly associated with worse survival, an association first shown by Sørensen and colleagues who analyzed data from more than 27,000 VTE patients in the Danish National Patient Registries [19]. Patients in whom cancer and VTE were diagnosed concurrently were compared to those with a cancer diagnosis without VTE, matched by cancer type, age, sex and year of diagnosis. Patients with VTE at the time of cancer diagnosis had a significantly higher 1-year mortality rate (HR 2.5, 95% CI 2.3–2.7). The association between VTE and mortality was also studied in the CATS cohort, a prospective observational cohort study comprising 1685 cancer patients [20]. During the 2-year study period, VTE occurred in 145 (9%) patients, of whom 79 (55%) died during follow-up, compared to 647 (38%) of those who did not develop VTE (HR 3.0, 95% CI 2.4–3.8). Although these studies showed that VTE is strongly correlated with mortality in cancer patients, it is unlikely that this association is causal. This is underscored by several randomized controlled trials which compared primary thromboprophylaxis to observation or placebo in cancer patients. These studies did show a reduction in VTE risk in those receiving thromboprophylaxis, but no significant difference in all-cause mortality (HR 0.93, 95% 0.8–1.1) [21,22]. Similarly, no difference in mortality was observed in studies evaluating LMWH in cancer without an indication for anticoagulation [23]. It is likely that the association between VTE and increased mortality risk merely reflects the prothrombotic state in patients with aggressive or progressive cancer, which can be caused by upregulation of procoagulant factors in tumor cells, such as tissue factor [24,25].

Nonetheless, VTE is often referred to as the second leading cause of death in cancer patients [26,27,28,29,30], which is mainly based on a study by Khorana and colleagues in which cause of death was assigned by treating physicians in 4466 cancer patients who had initiated chemotherapy [31]. However, the detailed breakdown of the study results shows that VTE may be less frequently fatal than often assumed. The most frequent cause of death was cancer progression (*n* = 100; 71%), followed by infection (*n* = 13; 9.2%), arterial thromboembolism (ATE) (*n* = 8; 5.6%), VTE (*n* = 5; 3.5%), unknown cause of death (*n* = 5, 3.5%), and death due to other causes (*n* = 9; 6.4%). The combined risk of arterial and venous thromboembolism is the second leading cause of death (*n* = 13; 9.2%), while the risk of fatal VTE, however, was lower than that of infectious disease, arterial thromboembolism, and death due to other causes. Moreover, the absolute risk of fatal VTE in this cohort was only 0.11%, which is in line with the fatal VTE rate of 0.5% and 0.6% in the control groups of the large FRAGMATIC and SAVE-ONCO trials [32,33].

Although there is a strong correlation between VTE and mortality in cancer patients, it remains unclear how often VTE directly results in death. Studies in which fatal VTE is ascertained by autopsy data are scant. Based on the currently available data, it appears that the absolute risk of fatal VTE might be not as high as often assumed. Consequently, the benefit of thromboprophylaxis will rely more on preventing VTE-related morbidity, decreased quality of life, and costs associated with VTE.

### 2.2. Morbidity and Quality of Life

When evaluating the impact of VTE on patients’ morbidity or quality of life, it is important to note that there is a wide spread in the severity of VTE. The severity of complications in cancer patients are commonly described according to the Common Terminology Criteria for Adverse Events (CTCAE) published by the National Cancer Institute [34]. In this document, the severity of adverse events is graded according to standardized definitions ranging from grade 1 (mild) to 5 (death). The severity of deep-vein thrombosis is graded as moderate (grade 2). Pulmonary embolism is graded as severe (grade 3), as life-threatening (grade 4) in case of hemodynamic instability, or as fatal (grade 5) in case of a fatal pulmonary embolism. Since deep-vein thrombosis and the majority of pulmonary embolism (e.g. incidental or subsegmental) do not cause hemodynamic instability or result in death, most VTE cases are classified as a grade 3 adverse event or lower. Nonetheless, in the non-cancer population, it is well recognized that VTE of all grades of severity are associated with substantial morbidity and has a negative impact on quality of life [35].

Few studies have evaluated the impact of VTE on quality of life in cancer patients. Lloyd and colleagues assessed changes in quality of life associated with recurrent VTE in patients in the CATCH trial, a randomized controlled trial that compared the efficacy and safety of tinzaparin with warfarin in 900 cancer patients with acute VTE during seven months of follow-up [12]. Each month, patients were asked to fill in EQ-5D questionnaires, which assesses health in five domains: mobility, self-care, usual activities, pain/discomfort, and anxiety/depression [36]. The influence of recurrent VTE on quality of life was estimated in a model that compared quality of life in those with recurrent VTE to that of a reference case, i.e. a male from Western Europe with symptomatic deep-vein thrombosis, an ECOG score of 1, and no distant metastasis at baseline. In this model, patients with recurrent VTE had a significantly lower quality of life compared to the reference case (0.57 vs. 0.65, *p* = 0.021). Hence, recurrent VTE during anticoagulant treatment appears to negatively influence quality of life in cancer patients, although potential cancer relapse coinciding with recurrence was not taken into account in this model. 

Marin-Barrera and colleagues evaluated the impact of primary VTE on the quality of life in a prospective cohort study comprising 128 cancer patients with VTE, and 297 cancer patients without VTE [37]. All patients completed general health-related and VTE-related questionnaires 1 month after inclusion, and indicated a significantly lower quality of life in cancer patients with VTE. These results should, however, be interpreted with caution since the groups were not matched, and substantial differences in baseline factors between the groups were observed which could be associated with quality of life, such as tumor type, performance score and cardiovascular disease history. In addition, several other factors associated with quality of life were not reported as time since cancer diagnosis, survival, specific cancer treatment, or whether patients received palliative or curative treatment. 

Several small qualitative studies have been performed to assess patients’ experience with cancer-associated VTE [10,11,38]. Seaman and colleagues interviewed fourteen patients with different types and stages of cancer who were previously diagnosed with pulmonary embolism or deep-vein thrombosis [10]. Patients mentioned that the diagnosis of VTE had a major impact on their lives, calling it “a distressing event with a profound impact on daily living”, sometimes even more so than the cancer itself. The symptoms of pain in patients with deep-vein thrombosis and dyspnea in pulmonary embolism were experienced as very burdensome on a daily basis. In a comparable qualitative study by Mockler and colleagues, ten cancer patients reported VTE as a significant setback during their cancer care and had difficulty coping with the event [11]. 

Overall, data on the influence of a first cancer-associated VTE on quality of life is limited. Findings from VTE patients without cancer cannot simply be extrapolated to cancer patients, because this latter group more often experiences anxiety, side effects of cancer treatment, and frequent hospitalizations. These factors make it complicated to estimate the impact of an additional VTE event on quality of life. More data on the physical and mental burden of VTE in cancer patients are needed to inform decision making about thromboprophylaxis, in which the harms of VTE and anticoagulation-related bleeding should be carefully balanced. 

### 2.3. Risk of Bleeding and Recurrent Venous Thromboembolism

Cancer patients with VTE are at high risk of both recurrent VTE and bleeding during long-term anticoagulant treatment. The incidence of recurrent VTE during the 6 months after the initiation of anticoagulant therapy for cancer-associated VTE ranges from 7 to 9% in large RCTs, while the risk of major bleeding ranged from 3% to 6% [39,40,41,42]. These risks are approximately two to three fold higher, compared to patients without cancer [18]. 

The consequences of recurrent VTE and major bleeding can be serious. In a recent systematic review of 14 RCTs, the case-fatality rate was 17% (95% CI, 14–21%) for recurrent VTE and 11% (95% CI 3–18%) for bleeding [43]. Since international guidelines recommend to treat patients with anticoagulation for as long as the cancer is active [8], patients remain at risk of bleeding throughout the course of their disease. As primary thromboprophylaxis can prevent VTE in cancer patients, long-term anticoagulation therapy and the concurrent bleeding risk can be avoided. 

### 2.4. Long-Term Sequelae

The long-term sequelae of VTE, including post-thrombotic syndrome in patients with deep-vein thrombosis and chronic thromboembolic pulmonary hypertension (CTEPH) in patients with pulmonary embolism, are also of clinical importance for cancer patients. Post-thrombotic syndrome is a common and burdensome syndrome that occurs in about 45% of non-cancer patients in the first 36 months after a deep-vein thrombosis [44]. It is associated with local pain, swelling, itching, cramps, or venous ulcers, and the severity can be assessed by using the Villalta score [45]. Compression stocking may reduce the risk of post-thrombotic syndrome, although there is controversy about their benefit [44]. Little evidence is available about post-thrombotic syndrome in cancer patients. A multicenter VTE registry in Japan, which included 3027 patients with VTE [46], showed that patients with active cancer had a higher risk of developing post-thrombotic syndrome (OR 3.6, 95% CI 2.3 to 5.8) than non-cancer patients, which might be due to the higher clot burden due to hypercoagulability in cancer patients. However, this increased risk of post-thrombotic syndrome in cancer patients was not observed in a large population-based study from the Netherlands including 1,668 patients (RR 0.8, 95% CI 0.4 to 1.4) [47]. Post-thrombotic syndrome was associated with a lower QoL in cancer patients enrolled in the CATCH trial [12]. 

CTEPH constitutes pulmonary hypertension caused by pulmonary embolism and can be associated with dyspnea, chest pain, and right-sided heart failure. The estimated incidence of CTEPH ranges from 0.5 to 5% in up to three years follow-up among different prospective studies in non-cancer patients [48,49], and the estimated three-year mortality is approximately 30% [48,50]. One small study assessed the incidence of CTEPH in 129 cancer patients, of whom only one (0.75%) was classified as “CTEPH likely” after six months. Although the presence of cancer could lower the threshold of suspicion for CTEPH, the low absolute risk probably does not justify routine screening for CTEPH in cancer patients. To our knowledge, no studies have been performed in quality of life in patients with CTEPH and cancer. 

CTEPH and post-thrombotic syndrome are serious long-term disease complications after VTE. Due to the dramatic improvement in cancer survival over the last decades, clinicians should be aware that an increasing number of cancer patients and survivors will experience these long-term sequelae of VTE [51]. 

### 2.5. Interference with Cancer Treatment

Cancer-associated VTE can result in delays or interruptions of cancer treatment, which was demonstrated in a retrospective cohort study of 534 patients with esophageal cancer undergoing neoadjuvant chemoradiation [13]. Among the 75 patients (14%) who developed a thromboembolic event, the median time until surgery was 11 days longer compared to those without such an event (47 vs. 36 days, *p* = 0.0004), although this did not result in a difference in 30-day mortality (1% vs. 2%, *p* = 0.9). In a retrospective cohort study of 2047 patients who underwent pancreaticoduodenectomy for pancreatic cancer, VTE in the 30-day postoperative period was associated with omission of adjuvant chemotherapy, also after adjusting for potential confounders (adjusted OR 1.9; 95% CI 1.1 to 3.4) [14]. 

### 2.6. Financial Burden

Several studies assessed the economic burden of cancer-associated VTE. A retrospective study from the United States evaluated costs associated with VTE in 6732 patients with lung cancer starting chemotherapy [15].In the patients with VTE, the average unadjusted costs in the entire 12 months were 33% higher ($84,187) than in those without VTE ($56,818; *p* < 0.0001). This difference persisted after adjustment for demographic variables, medical history, insurance type, and cancer supportive care treatments. Similarly, another study from the United States including 529 patients with cancer and deep-vein thrombosis reported an average subsequent hospitalization of 11 days and associated costs of $20,000 per patient (corrected for inflation) [16]. The mean hospital stay and costs increased to 18 days and $43,000 respectively when patients experienced in-hospital bleeding due to anticoagulation. These findings indicate that the diagnostic and therapeutic management of VTE and the potential bleeding complications in cancer patients is costly. Hence, primary VTE prevention could be beneficial from a financial perspective. 

### 2.7. Knowledge Gaps

Overall, it appears that little evidence is available concerning the burden of VTE in cancer patients. Although the increased risks of recurrence and bleeding during treatment are well-established, several knowledge gaps remain. More solid data are needed on the risk of fatal pulmonary embolism in cancer patients, the incidence of long-term VTE sequelae, and the impact of VTE on quality of life and on cancer treatment and whether this latter affects survival. Nonetheless, it is likely that VTE has at least similar negative consequences as in the general population, which should be taken into account when considering primary prevention of VTE in cancer patients. 

## 3. Primary Thromboprophylaxis in Ambulatory Cancer Patients

The negative consequences of VTE in cancer patients can, at least in part, be avoided with primary thromboprophylaxis, a strategy that is already universally accepted in other settings with a high risk of VTE, such as following major surgery, in pregnant women with thrombophilia or previous VTE, or in high-risk patients during hospital admissions. Levine and colleagues presented the first evidence in 1994 for a potential clinical benefit of primary thromboprophylaxis. In a randomized controlled trial of 311 metastatic breast cancer patients, those assigned to low-dose warfarin with an INR target of 1.3 to 1.9 had a significant 3.7% absolute lower risk of VTE than those receiving placebo during approximately six months of follow-up [52]. It was not until fifteen years later, a period during which low-molecular-weight heparins (LMWH) was established as standard therapy for cancer-associated VTE [39], that the PROTECHT randomized, placebo-controlled trial was published. This study randomized 1150 ambulatory cancer patients who initiated chemotherapy to either prophylactic subcutaneous nadroparin (3800 IU daily) or to placebo once daily [53]. After a median follow-up of 3.5 months, 15 of 769 patients allocated to nadroparin had developed VTE (2.0%) compared to 15 of 381 patients (3.9%) receiving placebo (*p* = 0.02). Despite this 1.9% absolute lower VTE risk with nadroparin, the authors proposed to focus future studies on cancer patients at high risk of VTE to decrease the number needed to treat (NNT).

Following this pivotal study, several other randomized trials with comparable designs and treatment regimens followed. These were subsequently summarized in a comprehensive Cochrane systematic review meta-analysis by Di Nisio and colleagues [21]. The pooled results of nine randomized trials that compared LMWH with placebo or observation demonstrated a lower risk of symptomatic VTE with LMWH (7.1%) than with placebo or observation (3.9%), translating into a relative risk of 0.54 (95% confidence interval (CI), 0.38–0.75). Given the corresponding substantial NNT of 30 patients to prevent one VTE as well as the increased tendency for major bleeding with thromboprophylaxis (RR, 1.4; 95% CI, 0.98–2.1), the authors concluded that more data were needed before implementation of routine primary thromboprophylaxis in ambulatory cancer patients could be justified. They also advised future studies to include patients at high-risk of VTE only. Other drawbacks of primary thromboprophylaxis with LMWH that limited its widespread use in practice include the inconvenience associated with long-term daily subcutaneous injections and the substantial costs of this therapy [54]. Indeed, 36% of patients in the nadroparin group enrolled in the PROTECHT trial and 29% of those receiving subcutaneous placebo injections, prematurely discontinued treatment [53], possibly reflecting the burden of daily subcutaneous injections in a vulnerable population. 

## 4. Prediction of Venous Thromboembolism in Cancer Patients

Several risk assessment tools for cancer-associated VTE have been introduced, which aim to reduce the NNT of primary thromboprophylaxis to prevent one VTE, by selecting high-risk patients only. The Khorana risk score, which was introduced in 2008, is currently the most widely known tool. It combines five clinically readily available variables to classify cancer patients initiating systemic anticancer treatment by their VTE risk [55]. Points are assigned for having a high or very high-risk primary tumor site (+1 or 2 points, respectively), pre-chemotherapy platelet count of 350 × 10^9^/L or higher (+1 point), pre-chemotherapy hemoglobin concentration lower than 6.2 mmol/L or use of erythropoiesis-stimulating agents (+1 point), pre-chemotherapy leukocyte count lower than 11 × 10^9^/L (+1 point), and a body mass index of 35 kg/m^2^ or higher (+1 point, Table 2). Based on the sum score, patients are classified as low risk (0 points), intermediate risk (1 or 2 points), or high risk (3 points or more). Although the score has been endorsed by various guidelines [6,56], the performance of the score remains a matter of debate. Conflicting results have been reported about the positive predictive value, the sensitivity is only modest, and the performance varies substantially among cancer groups, with lower discrimination in patients with lung cancer [57,58]. Two large prospective cohort studies, initiated to validate and derive VTE risk scores in cancer patients, both independently concluded that only Khorana score variable ‘primary tumor site’ was significantly associated with cancer-associated VTE, but not the other items, i.e., hemoglobin level, white blood cell count, platelet count, and body mass index [59]. Therefore, the question arises whether the Khorana score is merely a complicated score that selects cancer types with the highest risk, instead of cancer patients with the highest risk.

Improvements to the Khorana score were suggested by assigning additional points for chemotherapy [60], patient performance status [61], the laboratory biomarkers soluble P-selectin and D-dimer [62], cancer stage, vascular compression by the tumor, and prior VTE [63]. Others proposed to use a fibrin generation test [64], genetic risk factors [65], cardiovascular risk factors, and the use of anti-hormonal therapy [66]. However, in general, most scores are not yet externally validated, only marginally improved prediction [57], or include biomarkers that are not readily available in each medical institute, which precludes their clinical use [59]. Consequently, Pabinger and colleagues proposed a new simplified prediction model based only on primary tumor site and D-dimer as variables [59]. Discrimination was significantly improved compared to the Khorana score in both the derivation and validation cohort. Future intervention studies are, however, needed to validate these results, demonstrate clinical feasibility of routine D-dimer testing, and evaluate clinical benefit of the model. Tumor-specific risk scores were recently developed for hematologic malignancies [67,68] and gynecological malignancies [69]. Because neither of these scores have been externally validated nor have been acknowledged by the current guidelines, we will not further discuss these risk assessment tools.

## 5. Primary Thromboprophylaxis in Selected Cancer Patients

The PHACS trial, published in 2017 by Khorana and colleagues, was the first to evaluate thromboprophylaxis in a selected cancer population based on a high risk of VTE. In this randomized controlled trial, performed at seven sites in the United States and 1 in Canada, 117 cancer patients with a Khorana score of 3 points or higher were randomly allocated either to prophylactic subcutaneous dalteparin at 5000 IU once daily for 12 weeks or observation. There was no significant difference in the rates of recurrent VTE or major bleeding between the groups. Unfortunately, no firm conclusions could be drawn as the trial was prematurely closed due to poor accrual after a five-year recruitment period, possibly as a result of aversion among patients against daily subcutaneous injections and competition of other studies in this population [70]. However, the design of this trial, and the introduction of direct oral anticoagulants as attractive treatment for cancer-associated VTE [41,42], provided the basis for two subsequent randomized trials.

CASSINI was a randomized, double-blind, placebo-controlled, multicenter trial that enrolled ambulatory cancer patients with a Khorana score of 2 or higher [71]. Of the 1080 potentially eligible patients, 49 (4.5%) were excluded because of (asymptomatic) deep-vein thrombosis detected by protocol-mandated screening with duplex compression ultrasonography of both legs at baseline, and 190 (18%) were excluded for other reasons. The remaining 841 patients without screen-detected deep-vein thrombosis at baseline were randomized to either rivaroxaban 10 mg once daily or placebo once daily for up to 180 days. Throughout the trial, patients were screened for asymptomatic deep-vein thrombosis by compression ultrasonography every eight weeks. The primary efficacy outcome was the composite of any proximal deep-vein thrombosis in a lower limb, non-fatal or fatal pulmonary embolism, symptomatic upper extremity deep-vein thrombosis, and symptomatic distal deep-vein thrombosis in a lower limb. The most common cancer types were pancreatic (33%), gastric/gastroesophageal junctional (21%), and lung cancer (16%). In the intention-to-treat analysis, there was no significant difference in the occurrence of the primary outcome between the groups during the complete follow-up period; VTE occurred in 25 of 420 patients (6.0%) randomized to rivaroxaban and in 37 of 421 patients (8.8%) randomized to placebo (hazard ratio (HR), 0.66; 95% CI, 0.40–1.1; NNT 36). Notably, the study drug was prematurely discontinued by 44% of the patients in the rivaroxaban group compared and by 50% of those randomized to placebo. In a secondary analysis confined to the on-treatment period, the primary outcome occurred in 2.6% of patients allocated to rivaroxaban and in 6.4% allocated to placebo, translating into a statistically significant 60% relative risk reduction in VTE (HR, 0.40; 95% CI, 0.20–0.80; NNT 26). A two-fold higher major bleeding rate was observed in patients in the rivaroxaban group (2.0%) as compared to the placebo group (1.0%) (HR 2.0, 95% CI 0.59–6.5, number needed to harm (NNH) 100). All-cause mortality was comparable between groups: 20% in the rivaroxaban group vs 24% in the placebo group (HR, 0.83; 95% CI, 0.62–1.1). The authors concluded that the trial provided information on VTE incidence in patients with a high Khorana score, but did not establish the benefit of prophylactic treatment with rivaroxaban, because the primary outcome during the 180-day trial period was not statistically significant. Ultrasound screening for asymptomatic deep-vein thrombosis resulted in exclusion of a substantial number of patients from the trial, while such screening during the trial identified 15 incidental proximal deep-vein thromboembolic events of the lower extremities during follow-up accounting for roughly 25% of the events in the primary outcome. This approach, which does not represent common daily clinical practice, hampers the interpretation of the findings because the clinical consequence of such asymptomatic deep-vein thrombosis is unclear [72]. 

In the AVERT trial, published by Carrier and coworkers, ambulatory cancer patients with a Khorana score of 2 points or higher were randomized to either apixaban 2.5 mg twice daily or to placebo [73]. The primary outcome was the occurrence of VTE in the 180-day study period, comprising incidental or symptomatic proximal deep-vein thrombosis of an upper-, or lower limb, pulmonary embolism, and pulmonary-related death. Although the design was similar to that of the CASSINI trial, no routine screening for VTE was performed throughout this study. The most frequent cancer types were gynecologic cancer (26%), lymphoma (25%), and pancreatic cancer (14%). Of the 288 patients randomized to apixaban, 12 (4.2%) had developed VTE after 180 days of follow-up compared to 28 of 275 patients (10.2%) in the placebo group (HR, 0.41; 95% CI, 0.26–0.65; NNT 17). Apixaban significantly increased the risk of major bleeding, which occurred in 10 patients (3.5%) in the intervention group and in five patients (1.8%) in the placebo group (HR 2.00; 95% CI 1.0–4.0; NNH 59). The risk of clinically relevant non-major bleeding was numerically higher in the group treated with apixaban (7.3%) compared to the placebo group (5.5%), though not statistically significant (HR, 1.3; 95% CI, 0.89–1.8; NNH, 56). A sensitivity analysis restricted to the on-treatment period, showed a stronger relative reduction in VTE, while the absolute risk reduction was similar; the incidence was 1% in those treated with apixaban in this period, compared to 7.3% in those receiving placebo (HR 0.14, 95% CI 0.05–0.42, NNT 16). All-cause mortality during the trial was not significantly different between the two treatment groups (12.2% in apixaban vs 9.8% in the placebo group; HR, 1.3; 95% CI, 0.98-1.7). The AVERT trial was the first trial that showed that selecting high-risk patients for thromboprophylaxis may be clinically beneficial. Although the NNT of 17 is encouraging, it remains a question whether the benefit-harm ratio of prophylactic apixaban in this setting is perceived positive enough by oncologists, who need to consider this in the context of other disease complications, survival, and patients’ preference.

A recent systematic review aggregated data of the CASSINI and AVERT trial by performing a random effects meta-analysis of these two studies [22]. It is questionable whether a meta-analysis of just two studies is useful, also given the important differences in study design and cancer types included. Nonetheless, the summary risk ratio (RR) associated with the intervention in the intention-to-treat population during 6 months was 0.56 (95% CI, 0.35–0.89) for all VTE outcomes, and 0.58 (95% CI, 0.29–1.1) for symptomatic VTE. The summary RR for on-treatment major bleeding and CRNMB were 2.0 (0.80–4.8) and 1.3 (0.74–2.2), respectively.

It is important to emphasize that both trials considered patients with a Khorana score of 2 points or higher eligible for thromboprophylaxis, instead of the traditional threshold of 3 points or higher. A recent systematic review and meta-analysis, which evaluated the Khorana score in more than 34,000 cancer patients, estimated that 17% of all cancer patients have a Khorana score of 3 or higher and 47% a score of 2 or higher [58]. The estimated 6-month VTE incidence was lower in patients with a score of 2 or higher (8.9%; 95% CI 7.3–10.8) than in those with a score of 3 or higher (11%; 95% CI, 8.8–14). Hence, applying the lower threshold likely improved the feasibility of the CASSINI and AVERT trials, because more cancer patients were eligible for participation. However, this comes at the cost of a lower rate of VTE and higher NNT, as was confirmed in a pooled subgroup analysis of the AVERT and CASSINI trials; the pooled 6-month VTE incidence was 14.0% in patients with a Khorana score of 3 or higher receiving placebo versus 9.9% in the group with a score of 2 or higher [22]. The corresponding NNT was about 12 for cancer patients with a Khorana score of 3 or higher, but 17 for those with a score of 2 or higher. In the group of placebo recipients with a Khorana score of 2 points, the VTE incidence was 8.4% (NNT of 22). Whether thromboprophylaxis is justified for the latter group is debatable.

## 6. Where Do We Stand

Several international guidelines were recently updated after the publication of the CASSINI and AVERT trials. The guidance statement of the International Society on Thrombosis and Haemostasis (ISTH) now suggests the use of apixaban or rivaroxaban as primary thromboprophylaxis in ambulatory cancer patients starting chemotherapy with Khorana score ≥ 2 [7]. The guideline of the International Initiative on Thrombosis and Cancer (ITAC) recommends prophylactic rivaroxaban or apixaban for ambulatory cancer patients receiving systemic anticancer therapy at intermediate-to-high risk of VTE, identified by cancer type (i.e., pancreatic) or by a validated risk assessment model (i.e., a Khorana score ≥ 2) [74]. The latest guideline of the American Society of Clinical Oncologists (ASCO) is more prudent by stating that thromboprophylaxis with apixaban, rivaroxaban, or LMWH may be offered in cancer patients with Khorana score of 2 or higher [8]. All guidelines stress that the concomitant bleeding risk and patient preference should be taken into account. 

The CASSINI and AVERT trials showed the potential of long-term thromboprophylaxis with DOACs in selected cancer patients. Whether the results from these trials should be translated directly to clinical practice remains unsure. Both used the Khorana score for patient selection for thromboprophylaxis. The role of this score merits careful consideration before the results can be implemented into clinical practice, since its performance directly relates to the number needed to screen, absolute risk reduction, and NNT. 

**Figure 1:**
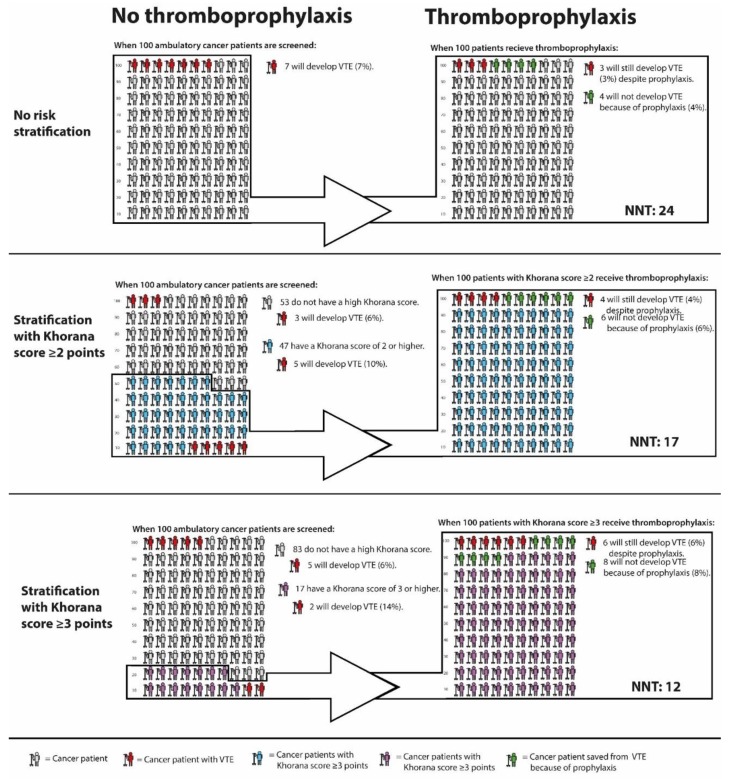
VTE incidence in cancer patients with and without thromboprophylaxis, stratified by Khorana score. Abbreviations: NNT, number needed to treat; VTE, venous thromboembolism. * Calculations based and extrapolated from Mulder et al; The Khorana Score For Prediction Of Venous Thromboembolism In Cancer Patients: A Systematic Review And Meta-Analysis [58], Carrier et al; Apixaban to Prevent Venous Thromboembolism in Patients with Cancer [73], and Li et al, Direct Oral Anticoagulant for the Prevention of Thrombosis in Ambulatory Patients with Cancer: A Systematic Review and Meta-Analysis [22].

Firstly, the sensitivity of the Khorana score is quite poor. When selecting patients with 3 points or more for thromboprophylaxis, 75% of all VTE events occur outside this high-risk group. When using a threshold of 2 points, approximately half of the total VTE events occur outside the high-risk group [58]. This means that the majority of cancer patients who will develop VTE do not benefit from risk stratification with the Khorana score, since they are not selected for thromboprophylaxis. The proportion of cancer patients classified as high-risk by a Khorana score cut-off value of 2 or 3 points, and the VTE incidence in these groups as observed in the AVERT trial, is graphically depicted in Figure 1.

Secondly, as the Khorana score is a pan-cancer prediction score, oncologists need to calculate the score for all their cancer patients to identify patients at high risk of VTE. This might be challenging given the daily time constraints experienced in the clinic, in particular for oncologists specialized in cancers at relatively low risk of VTE and a high number needed to screen, such as breast or prostate cancer. When a pan-cancer score is clinically used to select patients for primary thromboprophylaxis with DOACs, the premise is that the predictive performance of the score is similar for all cancer types. Previous studies, however, showed a substantial heterogeneity in the positive predictive value of the Khorana score across cancer groups [58]. Furthermore, a pan-cancer score should ideally have a similar benefit-risk trade-off of the treatment regimen for all cancer types. It has been acknowledged, however, that the efficacy and safety of DOACs in treatment of VTE may vary across cancer types [41,42,75,76]. For example, a substantial difference in the risk of VTE was observed between pancreatic and non-pancreatic cancer patients in the CASSINI trial. VTE incidence in the placebo group was 13% in those with pancreatic cancer and 6.7% in those with non-pancreatic cancer, resulting in a lower NNT for the former group. Conversely, the risk of bleeding may be greater for patients with gastrointestinal cancer than for those with other tumor types. For these reasons, an oncologist specialized in pancreatic cancer will likely be more interested in the safety and efficacy of thromboprophylaxis specifically for pancreatic cancer patients with a high-risk Khorana score than for other cancer types. However, other than for pancreatic cancer, data on safety and efficacy of primary thromboprophylaxis with DOACs are currently lacking for specific cancer types. More results of the CASSINI and AVERT trial stratified by cancer type are welcome, even if such analyses may be underpowered. More results of the CASSINI and AVERT trial stratified by cancer type are welcome, even if such analyses may be underpowered.

Lastly, it remains questionable whether the observed benefit-harm ratio of prophylactic DOACs in cancer patients with Khorana score 2 or higher justifies the large-scale implementation of primary thromboprophylaxis. As mentioned previously, the protocol-mandated deep-vein thrombosis screening applied in the CASSINI trial makes it difficult to translate the results to clinical practice. The NNT of 17 to prevent one VTE as observed in the AVERT trial is encouraging. However, the combined risk of major bleeding and clinically relevant non-major bleeding was 3.5% higher in the apixaban arm compared to placebo, corresponding to a NNH of 29. The NNT of prophylactic apixaban in cancer patients with and without risk stratification with the Khorana score is graphically depicted in Figure 1. Further uncertainties associated with DOACs are potential drug-drug interactions with chemotherapeutic agents [77], risk of bleeding during periods of thrombocytopenia, the optimal duration of thromboprophylaxis, and the benefit-harm ratio in patients with specific cancer types. For participants in the AVERT trial, the anticipation of minimum three months of chemotherapy was required, which presumably excluded patients receiving curative cancer treatment. Hence, it is unclear whether the trial results can be extrapolated to cancer patients receiving neoadjuvant chemotherapy.

## 7. Future Directions

**Figure 2:**
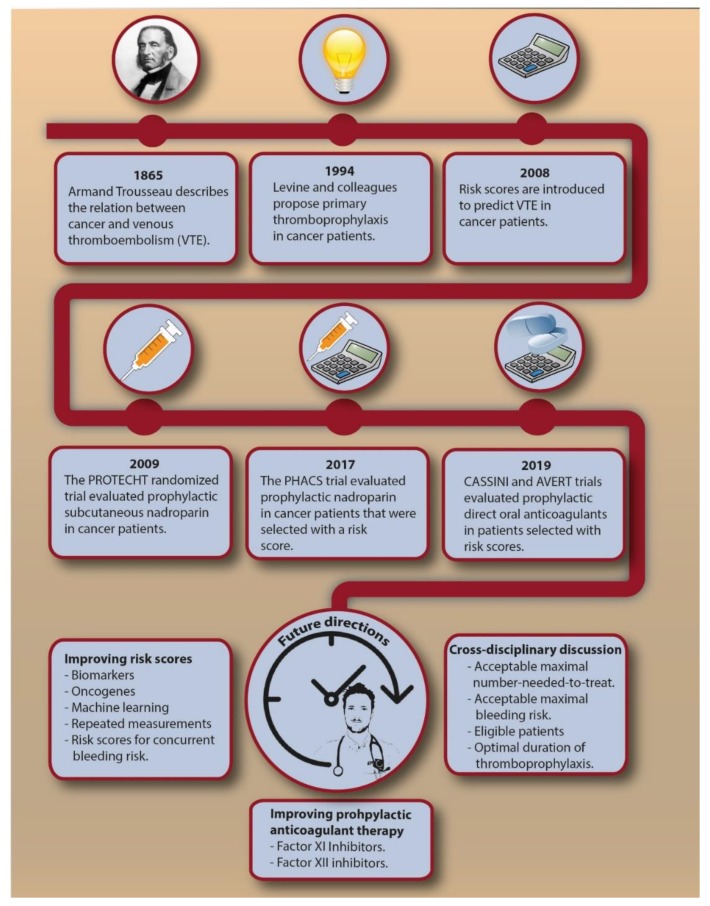
The chronological introduction of primary thromboprophylaxis in cancer patients and possible future directions. Abbreviations: NNT, number needed to treat; VTE, venous thromboembolism.

VTE is a frequent and potentially invalidating disease complication in cancer patients. Despite the recent and more remote advances (Figure 2), future steps in VTE prediction are needed to effectively reduce the burden associated with cancer-associated VTE. At present, we believe current evidence does not justify primary thromboprophylaxis in all cancer patients with a Khorana score of 2 or higher, which would have consequences for millions of patients worldwide. Additional randomized trials could strengthen the evidence and, when restricted to specific cancer types, address the uncertainty about the risk-benefit for specific cancer types. Improvements in VTE prediction in cancer patients are needed to lower the NNT to prevent one VTE associated with thromboprophylaxis. Most clinical prediction scores that were proposed as improvement to the Khorana score require further external validation. Several other clinical risk factors or biomarkers could ameliorate VTE prediction, such as serum platelet factor 4, citrullinated histone H3 as a marker for neutrophil extracellular traps, and extracellular vesicles bearing tissue factor, which showed promising results in smaller cohort studies [64,78,79]. Genetic risk factors for VTE have previously shown to be an important predictor for VTE in cancer patients [65], while specific tumor mutations also appeared to be associated with VTE [80,81]. This implies that genomic characterization of tumors could provide relevant information not only for cancer prognosis, but also for the risk of VTE. Repeated measurements of prediction scores should be explored to see whether continuously updated, dynamic risks are clinically helpful [82]. Machine learning could help in identifying new risk factors in large datasets to obtain accurate and precise personalized risk estimates [83]. From a pharmacological perspective, new agents for the treatment of VTE are currently explored, of which factor XI and XII inhibitors appear to be most promising [84,85,86]. If these agents prove to be safe and effective for the treatment of cancer-associated VTE, they could provide a basis for future thromboprophylactic strategies. More effort is needed in predicting the concurrent bleeding risk, which might help to reduce the NNH. Lastly, a cross-disciplinary discussion is needed to obtain consensus on an acceptable NNT and maximally tolerated bleeding risk.

## 8. Conclusions

In a broader perspective, one could question whether the development of an efficient pan-cancer VTE prediction score is feasible, given the large heterogeneity across cancer types in tumor biology, cancer treatment, and thromboembolic and bleeding risk. Possibly, prediction scores should be developed for specific cancer types to help effectively individualize strategies for primary thromboprophylaxis in cancer patients. Until then, thromboprophylaxis with DOACs should probably be restricted to cancer patients at very high risk of VTE and low risk bleeding after an informed discussion with the patient. 

## Figures and Tables

**Table 1:** Why VTE should be prevented in cancer patients.

**Short Term Consequences**
Increased mortality [9]
Morbidity caused by symptoms of pulmonary embolism or deep-vein thrombosis [10,11]
Reduced quality of life [12]
Interruption of cancer treatment [13,14]
Financial consequences [15,16]
**Long Term Consequences**
Post thrombotic syndrome [12]
Chronic Thromboembolic pulmonary hypertension [17]
Long-term bleeding risk [18]

**Table 2:** Khorana risk score.

Patient Characteristics	Risk Score
Site of cancer	
Very high risk (stomach, pancreas, brain)	2
High risk (lung, lymphoma, gynecologic, bladder, myeloma, testicular or kidney)	1
Prechemotherapy platelet count ≥ 350 × 10^9^/L	1
Prechemotherapy hemoglobin level < 6.2 mmol/L or use of red cell growth factors	1
Prechemotherapy leukocyte count > 11 × 10^9^/L	1
Body mass index ≥ 35 kg/m^2^	1
